# Comparing the Diagnostic Accuracy of Clinical and Radiological Measures in Hand Soft-Tissue Masses

**DOI:** 10.7759/cureus.12145

**Published:** 2020-12-18

**Authors:** Salman A Alzaidi, Qutaiba N Shah Mardan, Abrar Alotaibi, Mohamed Elmoursy

**Affiliations:** 1 Plastic and Reconstructive Surgery, King Faisal Medical Complex, Taif, SAU; 2 Plastic and Reconstructive Surgery, King Faisal Specialist Hospital and Research Centre, Riyadh, SAU; 3 Otolaryngology - Head and Neck Surgery, King Abdulaziz Specialist Hospital, Taif, SAU; 4 Plastic and Reconstructive Surgery, ME Clinics, Alexandria, EGY

**Keywords:** clinical diagnosis, diagnostic accuracy, soft tissue masses, radiological diagnosis

## Abstract

Introduction

Imaging modalities are imperative to aid in diagnosing hand soft-tissue tumors. Limited insight is available into the diagnostic accuracy of history and physical examination in comparison to radiological diagnosis.

Methods

In this retrospective analysis, data of patients with hand soft-tissue tumors that were surgically treated and diagnosed through biopsy were extracted; taking this as a reference, the sensitivity, specificity and positive and negative predictive values and likelihood ratios of the clinical approach and radiological tools were contrasted against each other.

Results

Data of a total of 34 patients were revised in this study. With a mean age of 40.1 years, the most common anatomical area of the hand to be affected by the tumors was the index (n = 7, (18.9%); ganglion cysts were the most common tumor (n = 9, 26.5%) and magnetic resonance imaging was the most commonly used imaging modality (n = 24, 70.6%). Clinical diagnosis scored a sensitivity and specificity of 44.4% and 100% in ganglion cysts and 62.5% and 86.2% in giant cell tumors in contrast to the sensitivity and specificity of 66.7% and 100% for ganglion cysts and 50% and 90% for giant cell tumors scored by radiological modalities.

Conclusion

Clinical diagnosis could be non-inferior to radiological diagnosis, yet radiological examination remains a valuable adjunct to clinical examination. Larger scale, prospective studies are required before generalizing our results.

## Introduction

Despite representing only 2% of the total body surface area [1] and 1.2% of total body weight, hands account for 15% of all soft-tissue tumors [2]. Fortunately, 95% of these, excluding tumors arising from the skin, are benign [3]. Most tend to present early, occupying relatively superficial structures and rendering them readily visible and palpable. Thus, most of the hand tumors have a favorable prognosis. Malignant soft-tissue masses that originate from hands, however, are unique, comprising only 2% of all hand lesions [4,5].

Ganglion cysts, giant cell tumor of tendon sheath (GCTTS), lipomas, nerve sheath tumors, glomus tumors, and hemangiomas are the most frequent hand soft-tissue tumors [6]. A ganglion cyst is encountered in about 70% of hand swelling cases, making it the most common [7]. A variety of modalities can be used to make a diagnosis, including high-resolution ultrasonography (USG) and magnetic resonance imaging (MRI), which are important methods [2]. USG shows the most potential to differentiate tumors from pseudotumors and for lesion characterization, based on its nature and relationships [8]. USG is inexpensive, and it can be performed quickly. Based on the high contrast and spatial resolution, MRI is the cornerstone when the nature of the lesion, enhancement pattern, and exact location are investigated [9].

This study aims to explore the efficacy of soft-tissue tumor diagnostic tools because studies that demonstrate the effectiveness and accuracy of such tools are scarce. Because of this gap in comparing the accuracy of clinical assessment to radiology that is used in making a diagnosis [10], this study, in accordance with Standards for Reporting Diagnostic accuracy studies (STARD) guidelines [11], aimed to study the accuracy of clinically based diagnoses of soft-tissue masses in the hand.

## Materials and methods

In this retrospective analysis, data were gathered from the patients’ files from November 2017 to February 2019. Ethics approval was obtained from the King Fahad Armed Forces Hospital ethics committee. The inclusion criteria were as follows: all patients presenting with soft-tissue masses in the hand to the plastic surgery clinic in King Fahad Armed Forces Hospital, Jeddah, Saudi Arabia, and who underwent surgery and had their diagnosis confirmed through histopathology. All patients who did not undergo surgery and/or whose diagnosis was not established through histopathology were excluded from this study. The data collected included the following demographic characteristics: age, gender, mass site, and location (right or left arm), and clinical, radiological, and histopathological diagnoses. Radiological and histopathological assessment was performed by radiology and pathology consultants.

The data were analyzed using SPSS version 25 (IBM Corp., Armonk, NY). Frequencies were calculated for all variables and measures of diagnosis accuracy were extrapolated. These included the following: sensitivity, specificity, positive predictive value, negative predictive value, positive likelihood ratio, and negative likelihood ratio.

## Results

Data from 34 patients were accessed, and more than half of the participants (67.6%; n = 25) were women, while 32.4% (n = 12) were men. The mean (±SD) age of the study population was 40.1 (±15.5) years (range: 5 to 70 years). Study participants’ demographic data are shown in Table 1. The highest percentage of patients with soft-tissue swellings was observed in patients who were 21 to 45 years old. However, subjects aged 0 to 20 years had the lowest percentage. 

**Table 1:** Demographic data.

Parameter	Percentage, value	Measures of dispersion	
Age	0-20 years of age 11.7% (n = 4)	M = 40.5 SD = 14.4; Minimum = 5, Maximum = 70	
	21-45 years of age 50% (n = 17)		
	46-70 years of age 38.2% (n = 13)		
Gender	Males 35.3% (n = 12)	-	
	Females 64.7% (n = 22)	-	
Left-sided lesion	50% (n = 17)		-
Right-sided lesion	50% (n = 17)		-

The distribution of lesions across the upper limbs is shown in Table 2. The lesions were located across the hand, most commonly in the index, thumb, and ring finger (18.9%, n =7; 16.2%, n = 6; and 13.5%, n = 5, respectively). Additionally, 2.9% (two patients) of the observed lumps were located in the third metacarpal region. Both sides had an equal (50%, n = 17) distribution in terms of dexterity or sinistrality. 

**Table 2:** Distribution of lesions across the upper limb.

Site	Percentage, value
2^nd^ digit	20.5% (n = 7)
1^st^ digit	17.6% (n = 6)
4^th^ digit	14.7% (n = 5)
3^rd^ digit	8.8% (n = 3)
5^th^ digit	8.8% (n = 3)
Hand dorsum	8.8% (n = 3)
Hand palm	5.8% (n = 2)
Volar aspect of the wrist	5.8% (n = 2)
Dorsal aspect of the wrist	5.8% (n = 2)
3^rd^ metacarpal area	2.9% (n = 1)

As shown in Table 3, among the different types of tumors that were diagnosed through histopathology, ganglion cysts were the most common (26.5%, n = 9), followed by GCTTS (23.5%, n = 8) and glomus tumors (8.8%, n = 3). 

**Table 3:** Prevalence of different types of tumors diagnosed through histopathology. Note: Three cases have been excluded, as there was no available data about diagnosis through histopathology.

Diagnosis proven on histopathology	Percentage, value
Ganglion cyst	26.5% (n = 9)
Giant cell tumor	23.5% (n = 8)
Glomus tumor	8.8% (n = 3)
Lipoma	5.8% (n = 2)
Traumatic neuroma	5.8% (n = 2)
Schwannoma	5.8% (n = 2)
Calcified aponeurotic fibroma	2.9% (n = 1)
Chronic synovitis	2.9% (n = 1)
Fibroma	2.9% (n = 1)
Angiomyoma	2.9% (n = 1)
Myoblastoma	2.9% (n = 1)

Different radiographic modalities were used preoperatively, including MRI in 70.6% of the patients (n = 24), USG in 14.7% (n = 5), X-ray in 5.9% (n = 2), and bone scan for 2.9% (n = 1). The sensitivity of clinical examination of the ganglion cyst was 44.4%, while the specificity reached 100%. Clinical examination showed 62.5% sensitivity and 86.2% specificity for giant cell tumors. Imaging modalities, overall, showed 66.7% sensitivity and 100% specificity for ganglion cysts. For GCTTS, imaging techniques scored 50% in sensitivity and close to 90% in specificity. For MRI, which was the most frequently used imaging modality in this population, the sensitivity and specificity were both 100% for ganglion cysts. However, MRI showed 66.7% sensitivity and 85.7% specificity for giant cell tumors. For USG, the sensitivity and specificity were both 100% for ganglion cysts and GCTTS. Further details are shown in Table 4. 

**Table 4:** Ganglion cyst versus giant cell tumor PPV: Positive predictive value, NPP: negative predictive value, +LR:  positive likelihood ratio and -LR: negative likelihood ratio of ultrasound and magnetic resonance imaging in the most two prevalent conditions in the study sample.

Ultrasound	Ganglion cyst	Giant cell tumor
	PPV = 100%	PPV = 100%
	NPP= 100%	NPP = 100%
	+LR = 0	+LR = 0
	-LR = 0	-LR = 0
Magnetic resonance imaging	PPV = 100%	PPV = 57.1%
	NPP = 100%	NPP = 90%
	+LR = 0	+LR = 4.4
	-LR = 0	-LR = 0.02

## Discussion

It is important to clinically assess palpable hand lesions and have imaging guidance to assist in confirming the diagnosis. In a review involving 122 confirmed lipoma cases, physical examination alone was sufficient to obtain a correct diagnosis 85% of the time. Moreover, clinical diagnosis was found to match the accuracy of USG in diagnosing hand and wrist tumors, although it was not as good as MRI [12].

USG showed a sensitivity, specificity, and positive predictive value of 100% for ganglion cysts. USG was shown to be accurate in diagnosing ganglion cysts 75% of the time, but GCTTS was not as high, at 50% [10].

MRI is widely considered to be the imaging technique of choice for characterizing and defining lesions to determine the best therapeutic plan. It was the most frequently used diagnostic modality in the current study. Our data suggest that MRI can accurately identify ganglion cysts, based on the 100% sensitivity, specificity, and positive predictive value. Similarly, albeit on a larger scale, McKeon et al. investigated the accuracy of the pre-operative MRI-based diagnosis of soft-tissue masses in the forearm, wrist, and hand [13]. After reviewing the records of 144 patients, they concluded that MRI was the most accurate test, especially with ganglion cyst cases, achieving a sensitivity of 94.7% and a specificity of 94.4%. Overall, MRI had a sensitivity of 75% for soft-tissue tumors of the upper limb. For GCTTS, however, the situation is different. The sensitivity was limited to 66.7% and the specificity was higher, at 85.7%. This wide variation in MRI accuracy among different hand soft-tissue tumors is not exclusive to our study [13]. A report on MRI accuracy in 20 patients with histologically proven benign peripheral nerve tumors showed a sensitivity of 75%, with five out of the 20 MRIs suggesting another diagnosis [14]. Another study involving 42 patients with diagnosed glomus tumors who underwent MRI as part of their pre-operative work-up showed a sensitivity of 86% [15]. Thus, we can conclude that pre-operative MRI for soft-tissue masses in the hands is critical, provided that caution is taken not to blindly follow the pre-operative diagnosis that is suggested by MRI analysis alone. Clinical examination may miss early cases of glomus tumors, especially subungual tumors, and thus, a decision based on a combined clinical examination and MRI would be optimal [16]. 

Several factors limit the generalizability and conclusions that can be drawn based on this study. In addition to the retrospective nature of the study, the small sample size decreases the strength of the study. Furthermore, diagnoses based on clinical sense are highly subjective to inter-personal variation in knowledge and experience.

## Conclusions

Relevant clinical examination plays an essential, and often overlooked, role, along with radiological investigations, in diagnosing hand soft-tissue tumors. Data in this study suggest that properly approaching a clinically based diagnosis of soft-tissue masses, specifically ganglion cyst and GCTTS, in the upper limbs could be non-inferior to making a diagnosis using imaging modalities. Until more conclusive data are available, the optimal practice should remain dependent on a combination of radiographic measures that are guided by clinical sense.
